# Pharmacological Modulation of Pupil Size in Presbyopia: Optical Modeling and Clinical Implications

**DOI:** 10.3390/jcm14176040

**Published:** 2025-08-26

**Authors:** Pablo De Gracia, Andrew D. Pucker

**Affiliations:** 1 School of Optometry, University of Detroit Mercy, Detroit, MI 48377, USA; degracpa@udmercy.edu; 2 Eminent Ophthalmic Services, LLC, 386 Riley Circle NW, Milledgeville, GA 31061, USA

**Keywords:** presbyopia, pupil size, clinical trials, pilocarpine, visual performance

## Abstract

Presbyopia is a ubiquitous age-related condition characterized by reduced near focusing ability due to lenticular stiffening. Pharmacologic agents such as pilocarpine have re-emerged as a less-invasive treatment option by inducing miosis and thereby enhancing depth of focus. However, the optimal pharmacologically induced pupil size that balances improved near vision with sufficient retinal illuminance remains undetermined. In this work, we present for the first time a direct integration of advanced theoretical modeling with a systematic synthesis of clinical trial outcomes to define the optimal target pupil size for pharmacologic presbyopia correction. We modeled visual performance using the Visual Strehl Ratio of the Optical Transfer Function (VSOTF) and convolved images of optotypes across a range of pupil diameters from 1.5 mm to 3.5 mm. This combined optical–clinical approach allowed us to quantitatively compare modeled image quality and depth of focus predictions with real-world clinical efficacy data from pilocarpine-based interventions. Simulations showed that smaller pupil sizes (1.5–2.5 mm) significantly extended depth of focus compared to standard multifocal optics while maintaining image quality within acceptable limits. These findings align with clinical trials of pilocarpine formulations, which commonly achieve post-treatment pupil diameters in the 2.0–2.5 mm range and are associated with clinically meaningful gains in near vision. Our analysis uniquely demonstrates that these clinically achieved pupil sizes closely match the theoretically optimal 2.0–3.0 mm range identified in our modeling, strengthening the evidence base for drug design and patient selection. These results reinforce the role of pharmacologically controlled pupil size as a central target in presbyopia management. By explicitly linking predictive optical modeling with aggregated clinical outcomes, we introduce a novel framework to guide future pharmacologic development strategies and refine clinical counseling in the emerging era of presbyopia therapeutics.

## 1. Introduction

Approximately 25% of the world’s population, which translates to 1.8 billion patients, has presbyopia [1]. The British Contact Lens Association’s (BCLA) Continued Learning Evidence-Based Academic Report (CLEAR) has defined presbyopia as a condition that “occurs when the physiologically normal age-related reduction in the eye’s focusing range reaches a point that, when optimally corrected for distant vision, the clarity of vision at near is insufficient to satisfy an individual’s requirements” [2]. While the full underlying mechanism resulting in presbyopia is still controversial, the most accepted mechanism is that age-related changes result in a stiffer crystalline lens, which decreases accommodative ability [3]. This lenticular deterioration not only results in worsening of uncorrected near vision, but presbyopia and its optical management have been associated with a negative impact on a patient’s quality of life [4,5]. While presbyopia has been historically corrected with spectacles, contact lenses, or refractive surgery [6], patients have indicated that they would prefer an eye drop over these options [4], which has propelled the field to develop topical pharmacological-based presbyopia treatments.

While miotic agents such as aceclidine, carbachol, and phentolamine mesylate have all been evaluated for the treatment of presbyopia [7,8,9], pilocarpine is the predominant pharmacological-based miotic presbyopia treatment described in the literature [10]. Topical 1% to 4% pilocarpine was originally introduced into the ophthalmic market as an intraocular pressure-lowering agent to treat glaucoma [11]; however, pilocarpine is rarely used in current clinical practice for glaucoma because there are other much more effective intraocular pressure-lowering agents (e.g., beta-blockers and prostaglandin analogs) [11]. Pilocarpine-based drops act in presbyopia via stimulation of muscarinic receptors on the iris sphincter and cause subsequent pupil constriction, which increases a patient’s depth of focus much like viewing through a pinhole [12,13]. This extended depth of focus improves a patient’s near vision, which consequently reduces a patient’s dependence on spectacles or contact lenses [14,15,16]. The other above-mentioned miotic drugs similarly treat presbyopia by increasing a patient’s depth of focus through pupil modulation [7,8,9]. Most available clinical data on pharmacologic miosis are derived from photopic and mesopic testing conditions, with fewer evaluations under scotopic lighting where functional performance may differ. Nevertheless, it is currently unclear what the ideal, real-world, pharmacologically induced pupil size should be to yield an optimal balance between near vision and retinal luminance. To address this gap, we present the first work to directly integrate advanced optical performance modeling (Visual Strehl Ratio of the Optical Transfer Function) with a structured synthesis of clinical trial outcomes for pharmacologic presbyopia treatment. This unique approach enables us to quantitatively compare predicted optimal pupil sizes with those actually achieved in real-world trials, providing novel translational insights that bridge theoretical optics and clinical practice. Thus, the purpose of this review and clinical opinion is to first describe the theoretically ideal pupil size and then put these data into context with past clinical studies, so the field can better develop treatments and educate patients.

## 2. Theoretical Pupil Size Targets

The influence of pupillary diameter on visual acuity has been of interest for the fields of optometry, ophthalmology, and vision science for more than a century [17,18]. Decreasing pupil size creates a smaller light pencil entering the eye, which in turn reduces the size of the diffusion circle and limits the amount of other irregularities, providing a clearer image and consequently better vision. Almost 50 years ago, Atchison et al. [19] showed the effect of pupil size on the visual acuity obtained with corrected and uncorrected myopia. Their findings demonstrated that corrected myopes achieved peak acuity with 2–3 mm pupils, while uncorrected myopes showed a strong dependence on pupil size, with optimal acuity often occurring at diameters smaller than 1 mm for refractive errors greater than 1.5 D. More recently, the 2016 study by Xu et al. [20] demonstrated that for presbyopic eyes, optimal pupil diameter varies with light level and viewing distance. Notably, reducing pupil size to 30–40% of its natural diameter enhanced near vision without significantly compromising distance acuity across most lighting environments. In another of their studies in 2019, Xu et al. [21] compared small-pupil and multifocal strategies for enhancing depth of focus in presbyopic and pseudophakic eyes. Xu et al. modeled a center-distance, dual-focus multifocal contact lens with a central distance-correction zone surrounded by concentric near-power zones and elevated positive spherical aberration (+1.0 µm or +1.5 µm for a 6 mm pupil) to replicate the optical profile of commercially available concentric-zone multifocal designs. At high light levels, where Weber’s law causes neural contrast sensitivity to become independent of retinal illuminance, small pupils (e.g., 1.0 to 3.0 mm) produced greater peak image quality and more effective depth of focus extension than the multifocal model based on small pupils. In low-light conditions, where decreased retinal illuminance due to pupil miosis reduced neural contrast sensitivity, small pupils (1.0 to 1.5 mm) consistently resulted in lower peak image quality across all levels of aberration. Under mesopic lighting, larger pupils combined with higher spherical aberration were most effective in enhancing depth of focus [21]. In 2023, we published a comprehensive evaluation of twelve commercially available multifocal contact lenses in a cohort of 65 eyes. A key takeaway from this study was that pupil size emerged as the most significant factor influencing optical performance across all tested designs. Subjects with pupil diameters between 2.4 mm and 3.0 mm consistently achieved the best optical quality through-focus, highlighting the critical role of pupil-mediated light distribution in the effectiveness of multifocal optics [22].

Pupil size steadily decreases with age, a phenomenon known as *senile miosis*. This age-related constriction is more pronounced for large pupil sizes occurring under dimmer lighting conditions than for smaller pupil sizes happening under bright light. Lazar et al. [23] provided detailed regression models showing how age affects pupil diameter across various luminance levels in natural environments. For instance, under high luminance conditions such as a sunny day (>1000 lux melanopic equivalent daylight illuminance [EDI]), pupil diameter was modeled with a slope of approximately −0.008 mm/year and an intercept of 2.57 mm. Based on this model, estimated pupil sizes for individuals aged 40, 45, 50, 55, 60, and 65 years are approximately 2.25 mm, 2.21 mm, 2.17 mm, 2.13 mm, 2.09 mm, and 2.05 mm, respectively. In contrast, under indoor lighting conditions (~10–100 lux melanopic EDI), where the slope steepens to −0.020 mm/year with an intercept of 4.32 mm, the estimated pupil sizes for the same age range are larger: 3.52 mm, 3.42 mm, 3.32 mm, 3.22 mm, 3.12 mm, and 3.02 mm, respectively. These findings underline the dual influence of age and environmental illumination on pupil dynamics in real-world settings [23]. Taken together, these regressions show that pupil diameter declines with age under both bright and indoor lighting, with a steeper age-related decrease at lower luminance.

To establish realistic and clinically relevant pupil sizes for the optical modeling presented in this study, we grounded our selection in both historical understanding and recent clinical evidence from pharmacological presbyopia treatments. As summarized in Table 1, contemporary pilocarpine-based therapies routinely induce pupil sizes in the range of 1.2 to 2.5 mm under photopic or mesopic conditions. For example, in studies evaluating 1.25% pilocarpine (VUITY^®^; Abbvie; North Chicago, IL, USA), pupil size decreased from approximately 3.2–3.4 mm at baseline to 1.8–2.0 mm within one hour of instillation under photopic light. Similarly, Farid et al. and Holland et al. [15,16] demonstrated post-treatment pupil sizes of 2.1–2.4 mm using lower concentrations of pilocarpine (0.2−0.4%) under mesopic and scotopic conditions. These pharmacologically induced pupil sizes correspond closely with the 2.0–3.0 mm range that prior studies—such as those by Xu et al. [20,21] and Atchison et al. [19]—have identified as optimal for balancing depth of focus and retinal illuminance in presbyopic eyes. Moreover, when modeling naked-eye optical performance, we also accounted for physiological pupil diameters typically observed in individuals aged 40 to 60 years under indoor lighting, which range between 3.0 mm and 3.5 mm. Accordingly, the pupil diameters selected for the modeling of small-aperture conditions—spanning from 1.5 mm to 3.5 mm—were chosen to reflect the functional pupil sizes achievable through both pharmacologic and natural modulation. Pupil diameters of 1.5–3.5 mm were selected to span pharmacologically achievable miotic pupils under photopic/mesopic conditions (treated state, ≈1.5–2.5 mm) and typical indoor physiological pupils in adults (untreated state, ≈3.0–3.5 mm), providing a clinically generalizable window across age groups. This range ensures clinical relevance by capturing the optical behavior of both treated and untreated presbyopic eyes. By anchoring our simulations in the empirically documented pupil sizes from recent clinical studies, we aim to provide translational insights into how pupil-modulating strategies affect through-focus image quality in real-world scenarios.

**Figure 1:**
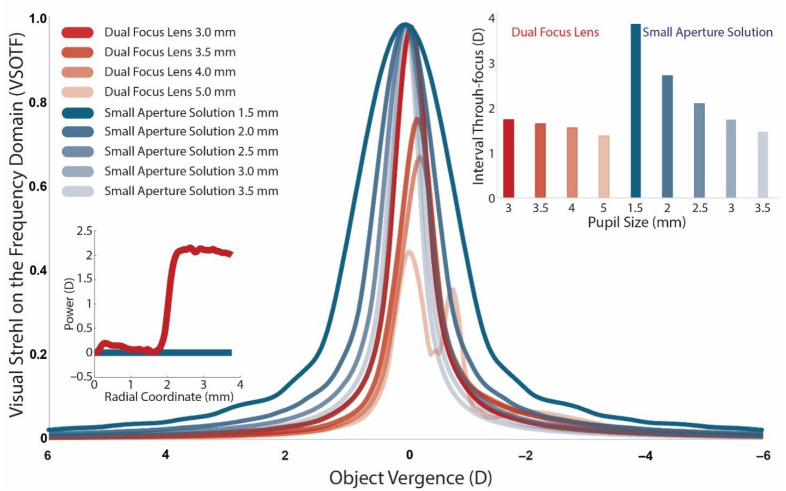
Visual Strehl Ratio on the Frequency Domain (VSOTF) through focus for dual focus and small aperture pupil conditions at various pupil sizes. The graph shows the through-focus VSOTF curves for a commercially available dual focus contact lens and a small aperture pupil model, each evaluated at four pupil diameters (3.0 mm, 3.5 mm, 4.0 mm, and 5.0 mm for a dual focus lens; 1.5 mm, 2.0 mm, 2.5 mm, 3.0 mm, and 3.5 mm for small aperture pupils). Peak optical quality is centered around 0 D vergence for all conditions. Smaller pupil sizes, particularly in the small aperture model (1.5 mm, 2.0 mm, 2.5 mm, and 3.0 mm), yield a broader depth of focus and higher VSOTF values over a wider range of defocus compared to larger pupils. The inset on the lower left corner represents the addition power for the modeled dual focus multifocal lens and a small pupil aperture. The inset bar graph quantifies the interval through focus—defined as the dioptric range over which VSOTF remains within an acceptable threshold—for each condition.

Using the methodology described in our prior study [22], we modeled optical quality through focus—measured by the Visual Strehl Ratio on the Optical Transfer Function (VSOTF) [32]—for a dual-focus commercial multifocal lens across typical indoor pupil sizes for individuals aged 40 to 60 years, which the power profile can be seen in Figure 1. We also computed VSOTF values for a naked eye with pupil sizes from 1.5 to 3.5 mm, achievable with the new pharmacological solutions for presbyopia available in the market. Figure 1 displays the resulting VSOTF curves, and the inset shows the interval through focus associated with acceptable visual acuity (VSOTF ≥ 0.12) [33] for each condition tested. These results highlight the influence of pupil size and design strategy on through-focus optical performance in presbyopic vision correction.

**Figure 2:**
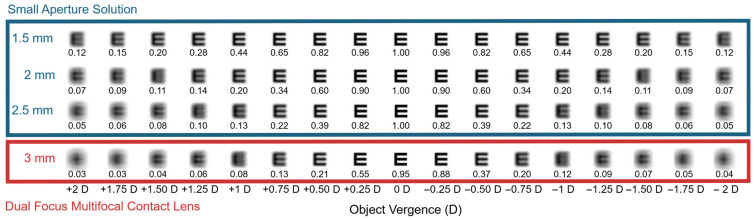
Convolved images of the letter “E” (20/100 visual acuity equivalent) are presented across a through-focus range from +2.00 D to –2.00 D of object vergence, simulating the optical performance of small-aperture solutions (top three rows) and a dual-focus multifocal contact lens (bottom row). The simulations incorporate pupil diameters of 1.5 mm, 2.0 mm, and 2.5 mm for the small-aperture model, and 3.0 mm for the multifocal contact lens condition. The numbers below each convolved letter represent the corresponding Visual Strehl ratio on the Frequency Domain (VSOTF).

To illustrate the optical quality associated with the conditions presented in Figure 1, Figure 2 shows convolved images of the letter “E” corresponding to a visual acuity of 20/100. These images depict through-focus performance across a vergence range from +2 D to –2 D for both three small aperture solutions of 1.5 mm, 2.0 mm, and 2.5 mm and a dual-focus contact lens of 3.0 mm [22]. For the convolved-image simulations (Figure 2), we assumed near-perfect dark adaptation—i.e., stable neural gain and contrast sensitivity at the modeled luminance—so that differences in image clarity reflect optical effects of pupil size rather than adaptation transients. However, in real-world low-light conditions, retinal mechanisms like dark adaptation and neural gain control may not fully compensate for reduced luminance. Additionally, increased photon noise under low-light levels can further degrade visual performance by reducing the signal-to-noise ratio of retinal responses. The bottom row simulates the through-focus response of a dual-focus multifocal contact lens with a 3 mm pupil, where the optical design includes discrete near and distance power zones. The result shows a narrower zone of acceptable visual quality (approximately +0.75 D to −0.75 D), with rapid degradation in clarity outside this interval. While multifocal designs may support simultaneous vision at discrete distances (as shown in Figure 1 for a pupil of 5 mm and a vergence value of around −1 D), small-aperture solutions provide a broader, smoother through-focus response.

As pupil diameter increases within the small-aperture conditions, the depth of focus narrows. The 1.5 mm, 2.0 mm, and 2.5 mm pupil sizes provide an extended range of usable image quality, maintaining legibility of the letter “E” of up to 4.00 D. These findings are consistent with theoretical expectations that smaller pupils reduce the blur circle diameter and aberration spread, enhancing depth of focus at the expense of retinal illuminance. A pupil size between 2.0 and 2.5 mm appears to offer the best compromise, preserving a meaningful extension of depth of focus while mitigating the decline in retinal illuminance that can impair visual performance under low-light conditions. While the simulations assume a near-perfect state of dark adaptation in which visual perception remains stable despite luminance reductions, real-world performance may be affected by incomplete compensation from retinal mechanisms such as dark adaptation and neural gain control. Moreover, under dim lighting, increased photon noise can further reduce the signal-to-noise ratio of retinal responses, potentially degrading image quality despite optical advantages. In real-world situations, a patient using a pharmacologic miotic agent to achieve a 1.5 mm pupil may report excellent near visual clarity under bright lighting but could struggle in dim environments such as restaurants or evening settings. While the optical benefits of extreme miosis are clear in simulations—providing a broad depth of focus and reduced blur—the accompanying reduction in retinal illuminance may result in darker images, decreased contrast sensitivity, and overall visual discomfort. By contrast, a pupil diameter in the 2.0 to 2.5 mm range may provide a more functional balance, delivering meaningful improvement in near vision while preserving sufficient luminance for acceptable image quality in everyday lighting conditions. A reduction from 3.5 mm to 1.5 mm decreases retinal illuminance to approximately 18% of baseline (≈0.74 log unit), which may not be fully offset by neural gain control in mesopic or scotopic environments. While our modeling identifies 2.0–2.5 mm as a functional balance for most viewing conditions, it is important to recognize that this conclusion is largely derived from photopic and mesopic data. In real-world low-light settings, incomplete neural adaptation and increased photon noise may reduce contrast sensitivity, and pupils smaller than ~2.0 mm may limit visual performance in activities such as night driving or reading in dim environments. This trade-off highlights the importance of considering not only optical metrics but also the neurophysiological and perceptual experience of the patient in low-light environments [20,21].

## 3. Pupil Sizes Achieved in Presbyopia Pilocarpine Clinical Studies

Benozzi et al. were among the first investigators to evaluate pilocarpine as a pharmacological treatment for presbyopia [24]. Benozzi et al. specifically proposed in 2012 the use of 1.0% pilocarpine plus 0.1% diclofenac twice a day (BID) for the treatment of presbyopia [24]. Diclofenac, which is a nonsteroidal anti-inflammatory drug (NSAID), was added to the formulation to reduce theoretical ocular inflammation that may be induced by pilocarpine [12,24]. Work by Farid et al. has subsequently found that the addition of an NSAID has little impact on near vision or side effects [15]. Benozzi et al. subsequently completed a series of retrospective case reviews with up to 10 years of follow-up and found that their 1.0% pilocarpine formulation was able to significantly improve near vision without impacting distance vision while inducing minimal side effects (Table 1) [24,25,26]. Benozzi et al.’s work did not evaluate pupil sizes [25].

More recent work from Waring et al. supported the first United States (US) Food and Drug Administration (FDA)-approved pilocarpine-based topical drop for presbyopia (1.25% pilocarpine; VUITY) [27]. Waring et al. specifically evaluated once-a-day (QD) treatment of presbyopia with 1.25% pilocarpine and found that treated participants were significantly more likely to meet the US FDA’s prespecified efficacy endpoint of an improvement of ≥3-line (15-letter) of visual acuity compared to participants treated with vehicle [27]. Work from Kannar et al. has since supported the use of 1.25% pilocarpine for BID dosing [14], and work from Lievens et al. has supported the use of 1.25% pilocarpine in the laser-assisted in situ keratomileusis (LASIK) and photorefractive keratectomy (PRK) populations [28]. Mousavi et al. has likewise compared two different formulations of 1.25% pilocarpine, with the authors finding that both formulations were able to significantly improve near visual acuity compared to the control treatment [10]. Waring et al. have lastly determined that while using 1.25% pilocarpine may have an impact on one’s ability to drive at night, likely due to the reduced amount of light into an eye with a constricted pupil, night driving performance was still at an acceptable level [29]. These 1.25% pilocarpine studies overall found a mean baseline pupil size range of 2.68–3.40 mm and a mean post-treatment pupil size range of 1.23–1.97 mm depending upon testing conditions (Table 1). These same studies found a pre- and post-treatment pupil size difference of 1.10 mm to 1.54 mm, depending upon lighting conditions at about 1 h post-treatment (Table 1).

Later work from Farid et al. and Holland et al. [15,16] has supported the approval of BID dosing of 0.4% pilocarpine (Qlosi; Orasis Pharmaceuticals; Ponte Vedra, FL, USA). Farid et al. specifically determined that 0.4% pilocarpine was the minimum concentration needed to produce a clinically meaningful improvement in near visual acuity (0.2% pilocarpine did not meet the visual acuity endpoint) [15]. Holland et al. later reported on two phase 3 trials that confirmed that 0.4% pilocarpine was able to produce a clinically meaningful improvement in near visual acuity while avoiding a negative impact on distance vision [16]. These 0.4% pilocarpine studies overall found a mean baseline pupil size range of 3.02–3.36 mm and a mean post-treatment pupil size range of 2.12–2.3 mm depending upon testing conditions (Table 1). These same studies also found a pre- and post-treatment pupil size difference of 0.90 mm to 1.10 mm, depending upon lighting conditions at about 1 h post-treatment (Table 1). When evaluating Farid et al.’s [15] 0.2% pilocarpine data, there was a mean baseline pupil size range of 3.17–3.28 mm and a mean post-treatment pupil size range of 2.37–2.46 mm depending upon testing conditions (Table 1). These same studies also found that there was a pre- and post-treatment pupil size difference of 0.80 mm to 0.82 mm, depending upon the drug formulation, at about 1 h post-treatment (Table 1).

Published non-pilocarpine-based studies have included work from Pepose et al. and Abdelkader et al. (Table 1) [7,8,9]. Pepose et al. specifically randomized presbyopic glaucoma participants to either 1% phentolamine mesylate or vehicle and found a significant post-treatment improvement in near visual acuity and a reduction in pupil size from 4.69 mm to 3.71 mm at 10–12 h post-treatment (net difference = 0.98 mm) [7]. Abdelkader et al. [8,9] similarly treated normal emmetropic, presbyopic participants with a combination of carbachol and brimonidine in a pair of randomized studies and found that carbachol was able to significantly improve post-treatment near visual acuity while also causing a significant post-treatment pupil size reduction (pre-treatment range = 4.30–4.77 mm vs. post-treatment range = 1.20–2.61 mm), which resulted in a net pupil size reduction of 2.11–3.10 mm at about 1 h post-treatment [8,9].

Therefore, if one considers that Farid et al. failed to find a clinically meaningful improvement in near visual acuity with 0.2% pilocarpine [15], the current data in the literature suggest that pilocarpine formulations should be at least 0.4% in concentration (Table 1), and miotic agents must cause at least a 0.90 mm constriction in pupil size to be able to obtain a clinically meaningful improvement in visual acuity as defined by the US FDA (≥3-lines of visual acuity). Nevertheless, a more formal, direct drug concentration comparison under the same lighting conditions is needed before any sort of definitive conclusion can be made on this topic.

## 4. Conclusions

Pharmacological modulation of pupil size has emerged as a promising strategy for presbyopia management, offering a less-invasive alternative to contact lenses and surgical interventions. Our modeling and literature synthesis reveal that pharmacologically induced miosis, particularly with pilocarpine-based treatments, can significantly enhance near visual acuity by increasing depth of focus through smaller pupil diameters. However, the balance between optical enhancement and sufficient retinal illuminance remains critical, especially under mesopic and scotopic conditions. The 2.0–2.5 mm target range represents a practical balance between optical quality and luminance in bright and moderate lighting. However, its suitability in scotopic environments requires further evaluation, and treatment should be individualized based on each patient’s visual demands and typical lighting exposure.

Our simulations and the current literature on the topic demonstrate that pupil diameters in the range of 2.0 to 2.5 mm, as achievable with currently available pharmacologic agents, offer meaningful extensions of depth of focus without severely compromising retinal illuminance. These findings were supported by both VSOTF-derived optical quality metrics and convolved image simulations. Importantly, our work emphasizes that while small-aperture solutions deliver smoother through-focus performance than multifocal optics, their effectiveness is closely tied to luminance conditions and baseline pupil size, which are influenced by both age and environmental lighting.

Reviewing clinical evidence, we observed that the majority of clinically effective pilocarpine-based treatments achieve a minimum of −0.9 mm pupil constriction to yield clinically meaningful improvements in near vision as defined by the US FDA. The efficacy threshold appears to reside in a pupil size reduction that brings the diameter into the 2.0–2.5 mm range under photopic or mesopic conditions. Additionally, the inclusion of lower pilocarpine concentrations in recent formulations (e.g., 0.4%) has demonstrated that substantial clinical benefit can be achieved while potentially minimizing side effects associated with excessive miosis. While use of these presbyopic drops results in reduced pupil sizes and subsequently improved near vision, their use is not without consequences. Presbyopic drops may result in adverse events such as site installation pain/discomfort/burning, headaches, blurred vision, conjunctival hyperemia, or punctate keratitis. However, these adverse events are typically short lived and mild if they do occur [14,15,16,26,27,29,30]. Furthermore, factors such as age, sex, systemic disease, obesity, and being pseudophakic are known to have an effect on pupil size [34], though how these factors impact the effectiveness of pharmacological treatments of pupil size and potential side effects has yet to be fully explored. Thus, additional research on these topics is still needed.

This body of work reinforces the concept that pupil size is not merely a passive outcome of pharmacological treatment but rather a central parameter that can be strategically targeted to optimize visual outcomes in presbyopic patients. Clinicians should be aware of the dynamic interplay between pupil size, depth of focus, and retinal luminance when prescribing these therapies, and future drug development should consider customizable or adaptive dosing strategies based on individual pupil dynamics.

In conclusion, the translation of optical modeling into clinical practice provides a robust framework for understanding and refining pharmacological approaches to presbyopia. Continued research bridging theoretical optics and real-world outcomes will be essential for personalizing treatments and setting new standards in presbyopic care.

## Figures and Tables

**Table 1:** Summary of pupil size in pilocarpine presbyopia treatment studies.

Study	Inclusion Criteria—Age (Years)	Inclusion Criteria—Refractive Error (D)	Sample Size	Treatment	Pupil Size (Baseline)	Pupil Size (Post-Treatment)	Significant	Post-Drop Application Time (Hours)	Lighting Conditions	Quality
	**Benozzi Method**									
Benozzi et al., 2012 [24]	45–50	Emmetropic Eyes	100	1.0% Pilocarpine + 0.1% Diclofenac, (BID)	Not Collected	Not Collected	N/A	Not Collected	N/A	Level 3
Benozzi et al., 2020 [25]	40–60	−0.50 D to +1.00 D with ≤1.00 D of cylinder	910	1.0% Pilocarpine + 0.1% Diclofenac, (BID)	Not Collected	Not Collected	N/A	Not Collected	N/A	Level 3
Benozzi et al., 2021 [26]	40–60	−0.50 D to +2.00 D with ≤1.50 D of cylinder	148	1.0% Pilocarpine + 0.1% Diclofenac, (BID)	Not Collected	Not Collected	N/A	Not Collected	N/A	Level 3
	**1.25% pilocarpine (VUITY^®^; AbbVie)**									
Waring et al., 2022 [27]	40–55	−4.00 D to +1.00 D with ≤2.00 D of cylinder	163	1.25% pilocarpine HCl, (QD)	3.40 ^#^	1.86 ^#^	N/T	1	Photopic	Level 1
			160	Vehicle, (QD)	3.21 ^#^	3.09 ^#^	N/T			
Kannarr et al., 2023 [14]	40–55	−4.00 D to +1.00 D with ≤2.00 D of cylinder	114	1.25% pilocarpine HCl, (BID)	3.21 ^#^	1.97 ^#^	Yes	1	Photopic	Level 1
			116	Vehicle, (BID)	3.04 ^#^	3.03 ^#^	No			
Lievens et al., 2024 † [28]	40–55	−4.00 D to +1.00 D with ≤2.00 D of cylinder	39	1.25% pilocarpine HCl, (QD)	Not Reported	Not Reported	N/A	Not Reported	N/A	Level 1
			41	Vehicle, (QD)	Not Reported	Not Reported	N/A			
Mousavi et al., 2024 [10]	40–60	≤1.00 D	45	1.25% pilocarpine HCl, (QD) ‡ (Unilateral Treatment)	2.68 ± 0.81	1.23 ± 0.58	Yes	1–2	Mesopic	Level 2
			30	1.25% pilocarpine HCl, (QD) (Unilateral Treatment)	2.78 ± 0.43	1.68 ± 0.50	Yes			
Waring et al., 2024 [29]	40–55	−4.00 D to +1.00 D with ≤2.00 D of cylinder	43	1.25% pilocarpine HCl, (QD)	Not Collected	Not Collected	N/A	Not Collected	N/A	Level 1
			43	Placebo	Not Collected	Not Collected	N/A			
	**0.4% Pilocarpine (Qlosi; Orasis Pharmaceuticals)**									
Farid et al., 2024 [15]	45–64	−4.50 D to +2.00 D with <2.00 D of cylinder	55	0.2% pilocarpine HCl (BID), days 8	3.17 ^#^	2.37 ^#^	Yes	1	Mesopic	Level 1
				0.4% pilocarpine HCl (CSF-1), (BID), days 15	3.02 ^#^	2.12 ^#^	Yes			
			53	0.2% pilocarpine HCl/0.006% diclofenac sodium (BID), days 8	3.28 ^#^	2.46 ^#^	Yes			
				0.4% pilocarpine HCl/0.006% diclofenac sodium (BID), days 15	3.13 ^#^	2.22 ^#^	Yes			
			58	0.006% diclofenac sodium (BID), days 8	3.46 ^#^	3.37 ^#^	No			
				0.006% diclofenac sodium (BID), days 15	3.42 ^#^	3.30 ^#^	No			
Holland et al., 2024 [16]	45–64	−4.50 D to +2.00 D with <2.00 D of cylinder	309	0.4% pilocarpine HCl (CSF-1), (BID)	3.36	2.30 ± 0.59	Yes	1	Scotopic	Level 1
			304	Vehicle, (BID)	3.51	3.43 ± 0.99	No			
	**Additional Pilocarpine Formulations**									
Vargas et al., 2019 [30]	41–65	−0.50 D to +1.50 D with <1.50 D of cylinder	117	0.247% pilocarpine + 0.78% phenylephrine + 0.09% polyethyleneglycol + 0.023% nepafenac + 0.034% pheniramine + 0.003% naphazoline, (QD)	3.30	3.05	Yes	2	Photopic	Level 2
Tripathi et al., 2024 [13]	40–55	Not Reported	120	2.0% pilocarpine	2.34 ± 0.37	1.57 ± 0.34	Yes	0.75	Photopic	Level 2
	**Charbachol Formulations**									
Abdelkader et al., 2015 [8]	43–56	±0.25 D (spherical equivalent) with ≤0.25 D of cylinder	30	2.25% Carbachol + 0.2% Brimonidine, (QD)	≥50 Years: 4.77 <50 Years: 4.72	≥50 Years: 2.50 <50 Years: 2.61	Yes Yes	1	Not Reported	Level 1
			18	Placebo	≥50 Years: 4.57 <50 Years: 4.80	≥50 Years: 4.48 <50 Years: 4.78	No No			
Abdelkader et al., 2016 [9]	42–58	±0.25 D (spherical equivalent) with ≤0.25 D of cylinder	10	3.0% Carbachol + 0.2% Brimonidine, (QD)	4.30 ± 0.50	1.20 ± 0.30	Yes	1	Not Reported	Level 1
			10	3.0% Carbachol, (QD) followed by 0.2% Brimonidine, (QD)	4.30 ± 0.50	1.90 ± 0.30	Yes			
			10	3.0% Carbachol, (QD)	4.30 ± 0.50	2.80 ± 0.50	Yes			
			10	0.2% Brimonidine, (QD)	4.30 ± 0.50	3.95 ± 0.50	No			
	**Phentolamine Mesylate Formulations**									
Pepose et al., 2021 [7]	≥18 Years ^δ^	Not Reported	19	1.0% Phentolamine Mesylate, (QD)	4.69 ± 0.95	3.71 ± 0.81	Yes	10–12	Photopic	Level 1
			20	Vehicle	Non-Significant Change		N/A			

N/A = Not Applicable; N/T = Not formally tested with a statistical analysis; † Sub-analysis of laser-assisted in situ keratomileusis (LASIK) and photorefractive keratectomy (PRK) participants from larger clinical trial; ‡ = Generic pilocarpine formulations; # Value extracted from a Figure with ImageJ 1.53m (https://imagej.net/ij/; Accessed on 16 June 2025); δ = Presbyopic glaucoma subjects. All studies included in the Table were graded based upon the level of evidence provided [31]. Level 1 evidence included high-quality randomized clinical trials. Level 2 evidence included well-designed controlled trials without randomization, well-designed cohort studies, or well-designed case–control studies. Level 3 evidence included case reports, descriptive studies, or expert opinions.
